# Veterinary Medical Association of New York County

**Published:** 1897-06

**Authors:** Robert W. Ellis

**Affiliations:** Secretary


### SOCIETY PROCEEDINGS.

### VETERINARY MEDICAL ASSOCIATION OF NEW YORK COUNTY.

THE regular monthly meeting of the Association was called to order by President Huidekoper, at eight o'clock sharp on the evening of April 7, 1897, at the Association rooms in the Academy of Medicine. On roll-call the following members responded: Drs. C. C. Cattanach, J. S. Cattanach, J. S. Cattanach, Jr., Delaney, Ellis, Farley, Gill, Huidekoper, Hanson, Loomes, O'Shea, and Robertson. The minutes of the previous meeting were read and approved.

The Board of Censors (Dr. Gill, Chairman) reported favorably on the application for honorary membership of Dr. W. Herbert Lowe, of Paterson, presented by members Ellis, Hanson, and Ryder. Moved and seconded that the report be accepted and that Dr. Lowe be declared an honorary member of the Association; carried.

Dr. Farley reported a case of injury to a mare by the introduction of a broom-handle into the vagina and uterus, evidently by malicious persons, which resulted in death soon after being seen by him. The Doctor reported the case, when seen, to the American Society for the Prevention of Cruelty to Animals, but no notice was taken of it by that society. Moved and seconded that Drs. Farley, Gill, and Hanson act as a committee of three to report at the next meeting on the case referred to by Dr. Farley and on the relations of the veterinarian to the Society for the Prevention of Cruelty to Animals; carried. Reports of other interesting cases by members, and free discussions followed, resulting in a motion by Dr. Delaney, seconded by Dr. Hanson, that Dr. Robertson read a paper, at the next meeting, on the transmission of diseases to the cat; carried.

The Judiciary Committee (Dr. O'Shea, Chairman) requested the Association to wait for a complete report of the legislative affairs until the next meeting, and stated that he feared the jury bill was doomed. He also read a communication from the Association's counsel relative to the Mulvey case, in which the counsel advised communicating directly with the County Clerk, as the error in allowing Mr. Mulvey to register is evidently his. Moved and seconded that the report be accepted; carried.

The Chair reported for the Publication Committee that all the details of the *Blue-Book* had at last been gone over, and that it was now ready for the printer. Moved and seconded that the report be accepted; carried.

The proposed amendment to the By-Laws was then read by the Secretary. Moved and seconded that the amendment be laid on the table; carried.

Moved and seconded that the meeting adjourn; carried.

THE regular meeting of the Association was called to order, May 5, at 8.45 P.M., at the Academy of Medicine, with the President, Dr. Huidekoper, in the Chair.

The following members responded to roll-call: J. S. Cattanach, Delaney, Ellis, Farley, Gill, Huidekoper, Hanson, Loomes, MacKellar, and Murphy.

The minutes of the previous meeting were read and, after slight corrections, approved.

The Board of Censors (Dr. Gill, Chairman) reported that the board had no business in hand.

The Committee on Dr. Farley's case and on the relation of the veterinarian to the American Society for the Prevention of Cruelty to Animals begged to report progress, as they had not had a meeting. Moved and seconded that the report be accepted; carried.

President Huidekoper then requested Dr. Cattanach to take the Chair, while he gave the members a very interesting and instructive "talk" on the subject of "castration standing," giving it as his opinion that the operation could not be properly performed standing, and exhibited specimens of testicles improperly removed by an operator in the standing position. A very interesting discussion followed by the members present.

President Huidekoper reported for the Publication Committee that they expected to have the *Blue-Book* out this month. Moved and seconded that the report be accepted; carried.

Dr. Gill read a communication from Dr. Salmon on the "Pasteur Monument Fund." Moved and seconded that the Chair appoint a committee of three with power to act on the communication from Dr. Salmon; carried.

Moved and seconded that the meeting adjourn; carried.

ROBERT W. ELLIS, D.V.S.,
Secretary.

